# Presence of salivary IgA anti-citrullinated protein antibodies associate with higher disease activity in patients with rheumatoid arthritis

**DOI:** 10.1186/s13075-020-02363-0

**Published:** 2020-11-23

**Authors:** Karin Roos Ljungberg, Emil Börjesson, Klara Martinsson, Jonas Wetterö, Alf Kastbom, Anna Svärd

**Affiliations:** 1 Division of Inflammation and Infection, Department of Biomedical and Clinical Sciences, Linköping University, Linköping, Sweden; 2 Center for Clinical Research Dalarna, Uppsala University, Uppsala, Sweden; 3 Department of Rheumatology in Östergötland, Linköping, Sweden

**Keywords:** Rheumatoid arthritis, Anti-citrullinated protein antibodies (ACPA), Mucosal immunity, Immunoglobulin A (IgA), IgA subclasses, Saliva

## Abstract

**Background:**

Circulating IgA anti-citrullinated protein antibodies (ACPA) associate with more active disease, but a previous study implied that salivary IgA ACPA is related to a less severe disease. Therefore, we aimed to characterize the IgA ACPA response in the saliva and serum in relation to clinical picture and risk factors among patients with rheumatoid arthritis (RA).

**Methods:**

RA patients (*n* = 196) and healthy blood donors (*n* = 101), included in the cross-sectional study “Secretory ACPA in Rheumatoid Arthritis” (SARA), were analyzed for ACPA of IgA isotype, and for subclasses IgA1 and IgA2 ACPA in paired saliva and serum samples using modified enzyme-linked immunosorbent assays (ELISA) targeting reactivity to a cyclic citrullinated peptide (anti-CCP). Cutoff levels for positive tests were set at the 99th percentile for blood donors. Antibody levels were related to clinical characteristics, radiographic damage, smoking habits, and carriage of HLA-DRB1/shared epitope (SE).

**Results:**

IgA ACPA in the saliva was found in 12% of RA patients, IgA1 occurred in 10%, and IgA2 in 9%. In serum, IgA ACPA was found in 45% of the patients, IgA1 in 44%, and IgA2 in 39%. Levels of IgA ACPA in the saliva correlated significantly with serum levels of IgA (*r* = 0.455). The presence of salivary IgA ACPA was associated with a higher erythrocyte sedimentation rate (ESR), 28-joint disease activity score, tender joint count, and patient global assessment at the time of sampling. None of the antibodies was associated with smoking, SE, or radiographic damage.

**Conclusion:**

Salivary IgA ACPAs were detected in a subset of RA patients in association with higher disease activity. This suggests that mucosal ACPA responses in the oral cavity may contribute to disease-promoting processes in RA.

## Introduction

Mucosal surfaces, rather than the joints, are proposed as sites of initial triggering events in rheumatoid arthritis (RA) development, particularly in anti-citrulline protein antibody (ACPA)-positive disease [1, 2]. ACPA can be detected in serum years before the onset of arthritis [3, 4], which implies that the autoimmune reaction starts years before the joints become involved. In clinical practice, IgG ACPA is often analyzed, but ACPA exists in several isotypes including IgA, and the latter has been identified in several mucosal fluids including saliva [5, 6].

IgA antibodies play important roles at mucosal surfaces, capable of both pro- and anti-inflammatory effects, e.g., opsonizing pathogens, neutralizing toxins, and inhibiting microbial adhesion to epithelia [7, 8]. IgA exists in two subclasses, IgA1 and IgA2, with different distributions in serum and mucosal fluids [9]. In serum, IgA1 dominates with a proportion of IgA1:IgA2 of 9:1, and in other secretions, the proportion is variable with higher proportions of IgA2 [9, 10]. Elevated relative amounts of IgA2 have been reported in colostrum, tears, nasal fluid, saliva, and intestinal lavage fluid [11–13].

In serum, monomeric IgA dominates, whereas in secretions, IgA is mainly dimeric. Dimeric IgA is produced by subepithelial plasma cells, and during the trans-epithelial transportation into the lumen, a protein chain, the secretory component (SC), is attached to form secretory IgA (SIgA) [8–10]. SIgA is mainly found in mucosal secretions, but may also be detected in small amounts in the circulation [14]. Several studies have highlighted local ACPA production at mucosal surfaces, possibly suggesting involvement in RA pathogenesis [6, 15–18]. We have identified circulating SC-containing ACPA (SC ACPA), which is associated with smoking and high inflammatory activity in early RA [18]. Whether or not levels of SC ACPA in the circulation reflect ACPA production in the oral cavity remains unknown.

Circulating IgA ACPA is found in 30–50% of RA patients and has been shown to associate with cigarette smoking as well as a more severe disease [5, 19, 20]. In contrast to IgG ACPA, IgA ACPA is not associated with the RA risk gene HLA-DRB1 shared epitope (SE) [20]. A possible inducer of ACPA production in RA is periodontal bacteria such as *Porphyromonas gingivalis* (*P. gingivalis*) which has the ability to citrullinate peptides in the oral cavity [2]. Antibodies to *P. gingivalis* have been demonstrated to associate with ACPA in RA patients [21], and oral priming by *P. gingivalis* has been shown to induce arthritis in rats [22]. IgA subclasses of rheumatoid factor (RF) have been investigated in sera and different mucosal secretions [23], where RF in serum and synovial fluid was found to be mainly of IgA1 subclass, whereas RF of IgA2 subclass was more prevalent in the saliva. In a previous pilot study, we reported the occurrence of IgA ACPA in the saliva in 22% of patients with RA, associating with less joint erosions [6].

In this study, we aimed to investigate salivary and circulating IgA ACPA antibodies, including subclasses, in relation to disease characteristics and risk factors in RA patients.

## Methods

### Study subjects and samples

One hundred ninety-six patients with established RA from the County of Dalarna, Sweden, were included in the cross-sectional, observational, “Secretory antibodies in Rheumatoid Arthritis” (SARA) study with enrolment 2012–2013. RA patients were randomly selected among planned follow-up visits at the Rheumatology Clinic, Falun Hospital, Sweden. Healthy blood donors (*n* = 101) were recruited as controls from the local blood donor center and referred to the Rheumatology Clinic for blood and saliva sampling. The ethics review board in Uppsala, Sweden, approved of the study, and all participants signed a written informed consent (Uppsala: 211/159). Participants were required to provide at least 0.5 mL of the saliva during the 10-min sampling time; otherwise, they were excluded from the study.

Paired saliva and serum samples were collected at the same visit at the Rheumatology Clinic. Participants were asked to restrain from eating, drinking other liquids than water, brushing teeth, or smoking 1 h before saliva sampling. Passive secretion was used for saliva sampling, i.e., the study participant leaned forward and drooled 10 min into a test tube placed on ice. After the disruption of mucus fibers by pipetting a few times, the saliva samples were centrifuged for 5 min at 5000*g*. Serum samples were centrifuged 5 min at 5000*g*. Serum and saliva samples were stored at − 80 °C until further analyses.

### Antibody analyses

Commercially available serum IgG-class anti-cyclic citrullinated peptide (anti-CCP) enzyme-linked immunoassays (ELISA) tests (CCPlus® Immunoscan, Euro Diagnostica AB, Malmö, Sweden) were modified to analyze IgA, IgA1, and IgA2 ACPA in the saliva and serum, as well as for serum SC and IgG ACPA. All samples were analyzed in duplicate, and reanalysis was carried out if the coefficient of variation between the duplicate samples was > 20%. The cutoff levels for a positive test of saliva ACPA (IgA, IgA1, and IgA2) were set at the 99th percentile among saliva samples from blood donors, and cutoff levels for serum ACPA (IgA, IgA1, IgA2, and SC) were set at the 99th percentile in serum among blood donors.

#### Antibody analyses in the saliva

The saliva was thawed at room temperature and centrifuged (15,000*g* at 4 °C for 10 min) to remove non-soluble material. The supernatant was diluted to a final concentration of 1:20. The secondary antibody in the salivary IgA ACPA assay (Polyclonal Rabbit Anti-Human IgA/HRP, DakoCytomation, Glostrup-Denmark) was diluted 1:200. Secondary antibodies for the subclass analyses (IgA1 antibody No. ABIN135642 and for IgA2 ABIN135642, Antibodies online, Aachen, Germany) were diluted 1:300. Incubation and washing were carried out according to the manufacturer’s instructions. Absorbance was read at 450 nm (Multiscan RC MOD 351, Labsystems, Helsinki, Finland).

To adjust for non-specific IgA adsorption, all samples were tested against a negative control peptide (cyclic arginine peptide, CAP, Euro Diagnostica AB). Anti-CCP and anti-CAP analyses were performed in parallel. Anti-CAP background levels were subtracted from anti-CCP values (delta optical density (OD)-measurements for anti-CCP and anti-CAP), and the cutoff values for a positive test were set to 0.78 for IgA ACPA, 0.70 for IgA1 ACPA, and 0.39 for IgA2 ACPA, corresponding to the 99th percentile of the 101 healthy blood donors. 

Total IgA in the saliva was analyzed with enzyme immunoassay tests from the IBL International (IgA Saliva ELISA, IBL International, Hamburg, Germany).

#### Antibody analyses in the serum

Serum samples were analyzed for IgG ACPA according to the manufacturer’s instruction (Euro Diagnostica AB), with cutoff set at 25 U/mL. Serum IgA and SC ACPA analyses were performed as previously described [5, 18]. In brief, for SC ACPA analyses, serum was diluted 1:25 and for IgA ACPA 1:100. As secondary antibodies, a polyclonal goat IgG anti-human secretory component (GAHU/SC/PO, NordicBiosite, Täby, Sweden) diluted 1:2000 in kit buffer was used in the SC ACPA assay (this secondary antibody does not differentiate between secretory IgA or secretory IgM, as it binds to the secretory component), and for the IgA ACPA assay, a polyclonal anti-human antibody diluted 1:2000 was used (Polyclonal Rabbit Anti-Human IgA/HRP, DakoCytomation, Glostrup, Denmark). High-leveled serum samples of IgA and SC ACPA were used to create standard curves. Absorbance was read at 450 nm (Multiscan RC MOD 351, Labsystems). Cutoff levels for positive serum tests of IgA ACPA were set to 25 arbitrary units (AU)/mL [5] and corresponding to the 99th percentile of the 101 healthy blood donors cutoff for SC ACPA was set to 72 AU/mL.

For IgA1 and IgA2 ACPA analyses, serum was diluted 1:50 and the subclasses were detected using anti-IgA1 and anti-IgA2 polyclonal mouse IgG anti-human Fc-region-specific antibodies, conjugated with HRP (IgA1/IgA2 Antibody, No.ABIN135642/ABIN135642, Antibodies online, Aachen, Germany). Secondary antibodies were diluted 1:500 in the IgA1 assay and 1:400 in the IgA2 assay. Seven-step serial dilutions of high-leveled serum samples were used as a calibrator, and the results were expressed as AU/mL. Incubation and washing were carried out as instructed by the manufacturer. Absorbance was read at 450 nm (Multiscan RC MOD 351, Labsystems). Cutoff limits for IgA1 ACPA were set to 27 AU/mL and for IgA2 ACPA 179 AU/mL.

Total IgA in serum was analyzed with a PEG-enhanced immunoturbidimetric method, using Siemens Atellica CH930 (Atellica CH Analyzer, Siemens Healthcare Diagnostics Inc. Tarrytown, NY, USA).

### Disease characteristics and risk factors

At inclusion, the participants answered a questionnaire regarding smoking habits [24]. At the time of sampling, the functional ability of patients was assessed using the Swedish version of the health assessment questionnaire (HAQ) [25], and disease activity was assessed using the 28-joint disease activity score (DAS28) [26]. RF status refers to the time point of RA diagnosis and was retrospectively obtained from medical records.

The prevalence of radiographic erosions was assessed from written reports by experienced radiologists in routine clinical care between 1982 and 2013. Results were dichotomized into having erosions or not.

### Genetic analyses

Genotyping of HLA-DRB1 was performed by Sanger sequencing (BGI Clinical Laboratories, Shenzhen, China). Shared epitope (SE) was defined as HLA-DRB1*01, *0401, *0404, *0405, *0408, *0409, *0410, *0413, *0416, *0419, *0421, or *10.

### Statistics

Statistical analyses were performed using SPSS v. 26.0 (SPSS, Chicago, USA), and two-tailed *p* values < 0.05 were considered significant. Antibody test results below the reference curve were given a value corresponding to half of the lowest detected value among patients and controls. Regarding saliva samples, negative delta values (i.e., when OD values for anti-CAP were greater than OD values for anti-CCP) were set to zero.

As IgG ACPA has been known to associate with poorer prognosis [27], comparisons of disease activity, radiographic outcome, smoking habits, and SE status, between IgA/IgA1/IgA2/SC ACPA-positive and ACPA-negative patients were performed only within the subgroup of IgG ACPA-positive patients, in order to avoid the confounding effect of IgG ACPA. Fisher’s exact test was used to test differences in proportions regarding smoking status, positive tests regarding different salivary, and circulating ACPAs (IgA, IgA1, IgA2, SC, and IgG) and SE. The Mann-Whitney *U* test was used to relate antibody status in the serum and saliva versus clinical variables, inflammatory markers, HAQ, and levels of other ACPAs. Spearman’s rho correlation coefficient (*r*) was used to test correlations between antibody levels in the saliva and serum. Linear regression analysis was used to evaluate associations between disease activity and levels of salivary IgA ACPA adjusting for levels of serum IgA ACPA, disease duration, and treatment. By including 196 RA patients of which 12% tested positive for IgA ACPA in the saliva, we achieved 80% power at alpha = 0.05 to detect a difference in DAS28 of 0.9 units between patients positive vs. negative for IgA ACPA in saliva.

## Results

### Occurrence of IgA ACPA and IgA ACPA subclasses in the saliva and serum

**Fig. 1:**
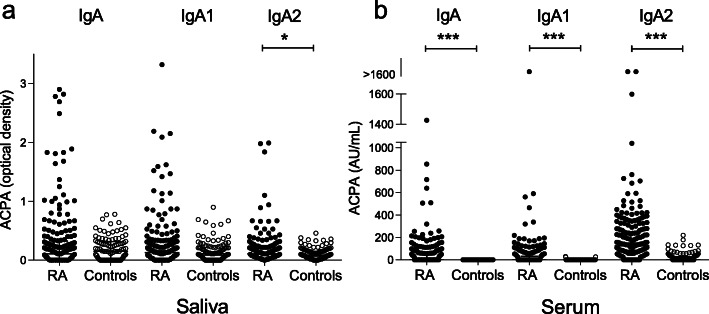
Distribution of IgA ACPA isotypes in **a** the saliva and **b** serum in RA patients and controls. ***p value < 0.001, *p value < 0.05

The 196 RA patients included in the SARA study had a mean disease duration of 12.2 years and 80% were women. Additional baseline characteristics of patients and controls are shown in Table 1. Levels of IgA, IgA1, and IgA2 of ACPA in the saliva and serum in patients with RA and controls are illustrated in Fig. 1.

**Table 1:** SARA baseline characteristics

	RA patients (*n* = 196)	Blood donors (*n* = 101)	***p*** value
**Characteristics**			
Women, *n* (%)	156/196 (80)	54/101 (53)	< 0.001
Age, mean (range)	63.9 (25–87)	48.6 (20–74)	< 0.001
Disease duration years, mean (range)	12.2 (0–57)	–	
Erosions, *n* (%)	109/193 (56)	–	
Biologics, *n* (%)	72/196 (37)	–	
Glucocorticoids, *n* (%)	52/196 (27)	–	
Methotrexate, *n* (%)	148/196 (76)	–	
Other csDMARD, *n* (%)	40/196 (20)	–	
**Risk factors**			
Ever smoker, *n* (%)	99/192 (52)	34/99 (34)	0.006
Current smoker, *n* (%)	15/192 (8)	10/99 (10)	0.514
Never smoker, *n* (%)	93/192 (48)	65/99 (66)	0.006
Cigarette pack-years, mean (SD)	13.9 (13.8)	8.3 (11.3)	0.009
Shared epitope carriage, *n* (%)	163/196 (83)	47/101 (47)	< 0.001
No allele, *n* (%)	34/196 (17)	54/101 (54)	< 0.001
One allele, *n* (%)	97/196 (50)	33/101 (33)	0.007
Two alleles, *n* (%)	65/196 (33)	14/101 (14)	< 0.001
**Antibodies**			
RF-positive, *n* (%)	145/195 (74)	–	
IgG ACPA			
Positive, *n* (%)	157/196 (80)	0/101 (0)	< 0.001
Level, median U/mL (SD)	444.1 (923.8)	0.1 (0.0)	< 0.001
IgA ACPA			
Positive, *n* (%)	88/196 (45)	0/101 (0)	< 0.001
Level, median AU/mL (SD)	1.3 (151.9)	1.3 (0.0)	< 0.001
Level total IgA in serum, median g/L (SD)	2.5 (1.4)	2.0 (0.7)	< 0.001
Level total IgA in saliva, median μg/mL (SD)	61.5 (85.8)	35.7 (24.7)	< 0.001

*Abbreviations*: *csDMARDs* conventional disease-modifying anti-rheumatic drugs, *RF* rheumatoid factor, *ACPA* anti-cyclic citrullinated peptides

#### Saliva

**Fig. 2:**
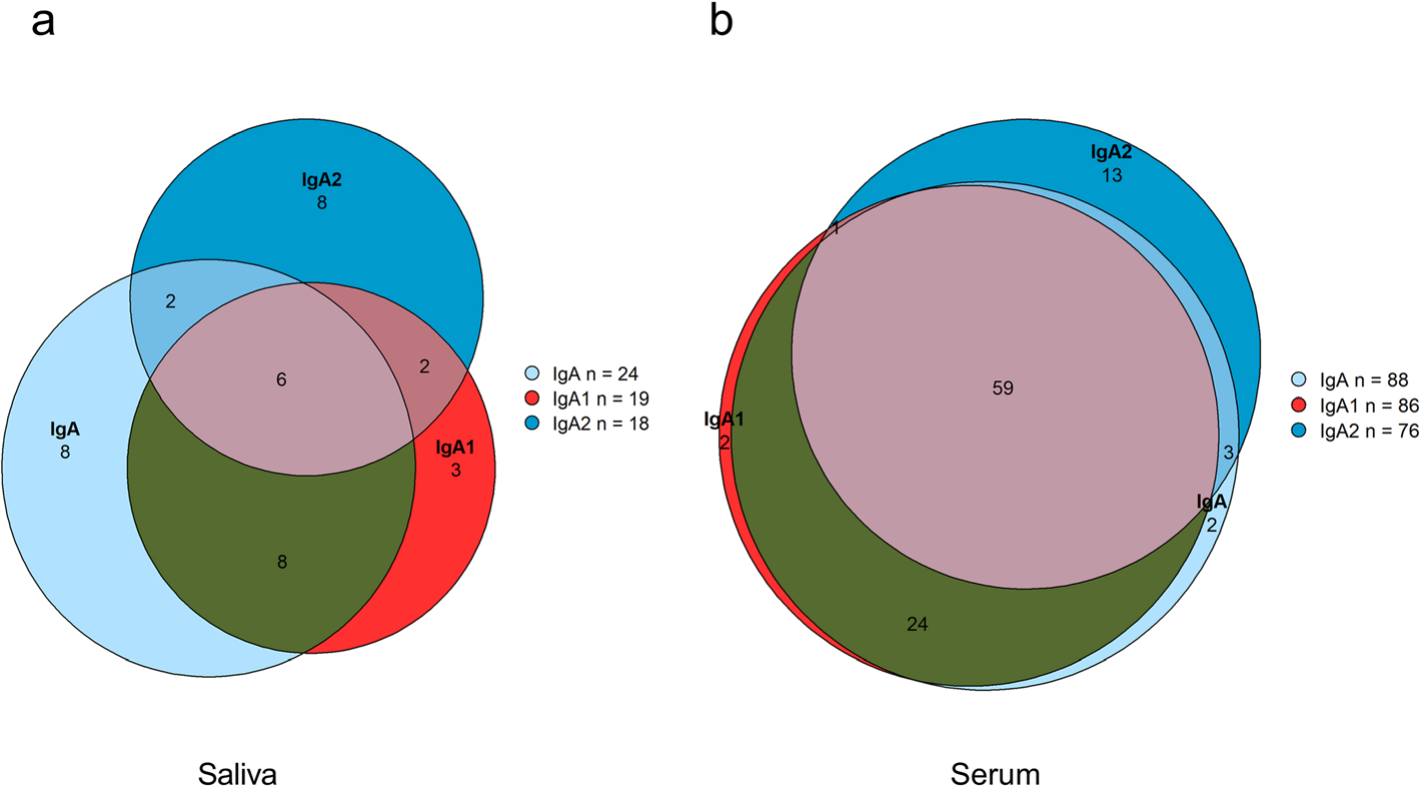
Venn diagram showing ACPA status of RA patients in **a** the saliva and **b** serum

In the saliva samples, 24 (12%) patients were positive for IgA ACPA, 19 (10%) for IgA1, and 18 (9%) for IgA2. Six saliva samples were positive for all tested IgA ACPA in the saliva (IgA+/IgA1+/IgA2+), 8 were positive for both IgA ACPA subclasses (IgA1+/IgA2+), 11 were positive for only IgA1 (IgA1+/IgA2−), and 10 were positive for only IgA2 (IgA1−/IgA2+) (Fig. 2a). In the saliva samples, 166 were negative for both subclasses (IgA1−/IgA2−).

#### Serum

IgG ACPA was detected in 157 (80%), IgA ACPA in 88 (45%), and SC ACPA in 42 (21%) RA patient sera. Serum IgA1 ACPA was detected in 86 (44%) of the patients and in 76 (39%) for IgA2 (no differences in the prevalence of IgA subclasses, *p* = 0.356). IgA subclasses as well as SC ACPA occurred predominantly in the IgG ACPA-positive subset of the patients. IgG ACPA co-occurred in 87 out of 88 (99%) IgA ACPA-positive patients, 85 out of 86 (99%) IgA1 ACPA-positive patients, 73 out of 76 (96%) IgA2 ACPA-positive patients, and all SC ACPA-positive patients (100%). Twenty-eight patients were positive for all tested ACPAs in serum (IgG, IgA, IgA1, IgA2, and SC). Sixty were positive for both IgA ACPA subclasses (IgA1+/IgA2+), 26 positive for only IgA1 (IgA1+/IgA2−), 16 positive for only IgA2 (IgA1−/IgA2+) (Fig. 2b), and 94 negative for both subclasses (IgA1−/IgA2−).

### Correlations of different ACPAs in the saliva and serum

Levels of IgA ACPA in the saliva were moderately correlated with serum levels of IgA (*r* = 0.455), IgA1 (*r* = 0.434), and weakly to the other serum ACPA isotypes; IgA2 (*r* = 0.277), SC ACPA (*r* = 0.29), and IgG (*r* = 0.342), all at the *p* < 0.001 level (Table 2).

**Table 2:** Correlations between different ACPAs in the saliva and serum

	Salivary IgA ACPA		Salivary IgA1 ACPA		Salivary IgA2 ACPA	
	*r*	*p* value	*r*	*p* value	*r*	*p* value
**Serum:**						
IgG ACPA	0.342	< 0.001	0.226	0.001	0.079	0.272
IgA ACPA	0.455	< 0.001	0.388	< 0.001	0.119	0.097
IgA1 ACPA	0.434	< 0.001	0.368	< 0.001	0.124	0.084
IgA2 ACPA	0.277	< 0.001	0.158	0.027	0.110	0.126
SC ACPA	0.290	< 0.001	0.194	0.007	0.026	0.722

*Abbreviations*: *ACPA* anti-cyclic citrullinated peptides, *SC* secretory component containing, *r* Spearman’s rho correlation coefficient

### Disease activity in relation to ACPA in the saliva and serum

Comparisons of disease activity measures were restricted to the serum IgG ACPA-positive patient subset (*n* = 157) as described in the “Methods” section.

#### Saliva

**Fig. 3:**
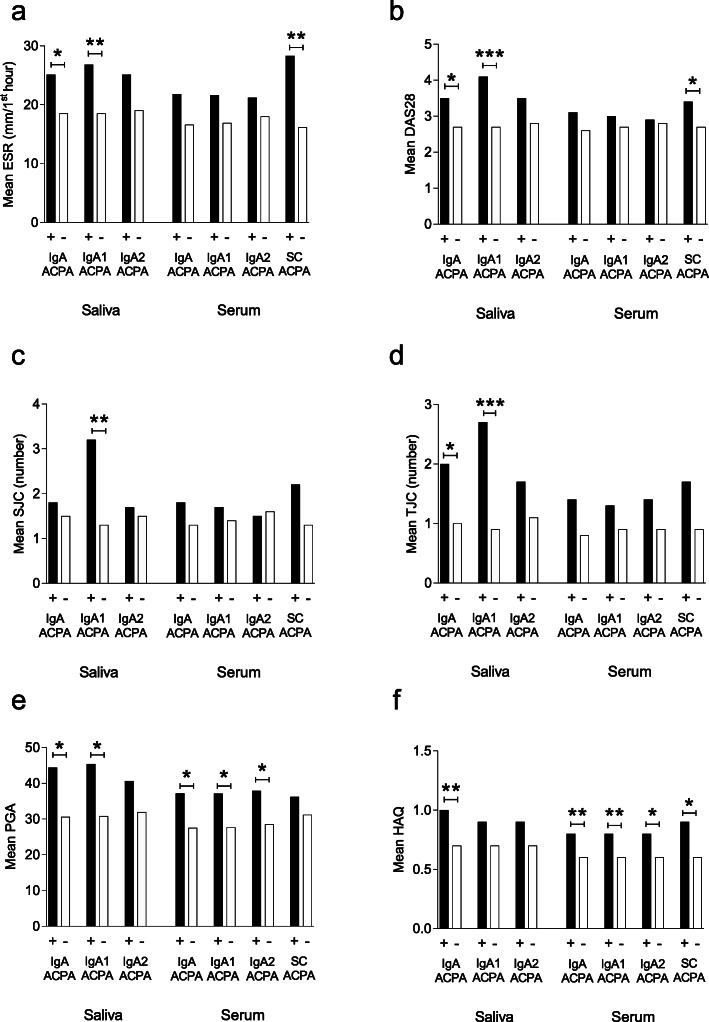
Antibody status versus disease characteristics at the time of sampling, regarding **a** erythrocyte sedimentation rate (ESR), **b** disease activity score 28 (DAS28), **c** swollen joint count (SJC), **d** tender joint count (TJC), **e** patient global assessment (PGA), and **f** health assessment questionnaire (HAQ). ***p value < 0.001, **p value < 0.01, *p value < 0.05

At the time of sampling, patients positive for IgA ACPA in the saliva had significantly higher erythrocyte sedimentation rate (ESR) (mean 25 vs. 19 mm/first hour, *p* = 0.031), DAS28 (mean 3.5 vs. 2.7, *p* = 0.04), and tender joint count (TJC) (mean 2.0 vs. 1.0, *p* = 0.039) compared to patients negative for IgA ACPA in the saliva. Also, patients positive for IgA ACPA in the saliva had higher HAQ (mean 1.0 vs. 0.7, *p* = 0.006) and patient global assessment (PGA) (mean 44 vs. 31, *p* = 0.03) (Fig. 3a–f). Similar significant differences were obtained regarding IgA1 ACPA, but not for IgA2 ACPA (Fig. 3a–f).

In linear regression analysis, salivary ACPA levels are associated with DAS28 (*p* = 0.016), and when adjusting for levels of serum IgA ACPA, the association was borderline significant (*p* = 0.071). DAS28 was associated with levels of salivary IgA ACPA also when adjusting for disease duration and treatment with biologic drugs and/or glucocorticoids at the time of saliva sampling (*p* = 0.021). When analyzing the impact of the secretory rate of the saliva (mL/min), there was no correlation between levels of salivary IgA ACPA and secretory rate (*r* = − 0.099, *p* = 0.217).

#### Serum

Serum IgA, IgA1, and IgA2 ACPA were all associated with significantly higher HAQ and PGA at the time of sampling compared to patients negative for the respective antibody. Patients positive for IgA ACPA in the serum had higher HAQ (mean 0.8 vs. 0.6, *p* = 0.005) and PGA (37 vs. 28, *p* = 0.014) than patients negative for IgA ACPA. Similar findings were obtained regarding IgA1 and IgA2 ACPA (Fig. 3a–f).

Patients positive for SC ACPA in the serum showed significantly higher levels of ESR (mean 28.3 vs. 16.2, *p* = 0.002), C-reactive protein (CRP) (mean 14.1 vs. 5.3, *p* < 0.001), DAS28 (mean 3.4 vs. 2.7, *p* = 0.011), and HAQ (mean 0.9 vs. 0.6, *p* = 0.029) (Fig. 3a–f).

### Radiographic outcome

#### Saliva and serum

Among patients who developed the erosive disease, the first radiographic erosion was detected at a mean time of 2.9 years (SD 6.0 years) after diagnosis. The presence of radiographic erosions did not differ significantly between groups according to saliva or serum IgA, IgA1, or IgA2 ACPA status (Supplementary Table 1). Neither did SC ACPA status in serum associate with radiographic erosions.

### Smoking habits and genetic disposition (shared epitope)

Smoking habits and carriage of SE in patients and controls are shown in Table 1. “Ever smoking” was significantly more common among RA patients compared to controls (52% vs. 34%, *p* = 0.006) while “current smoking” was not. SE was detected in 163 of 196 (83%) of the RA patients and in 47 of 101 (47%) of controls (*p* < 0.001).

#### Saliva

**Fig. 4:**
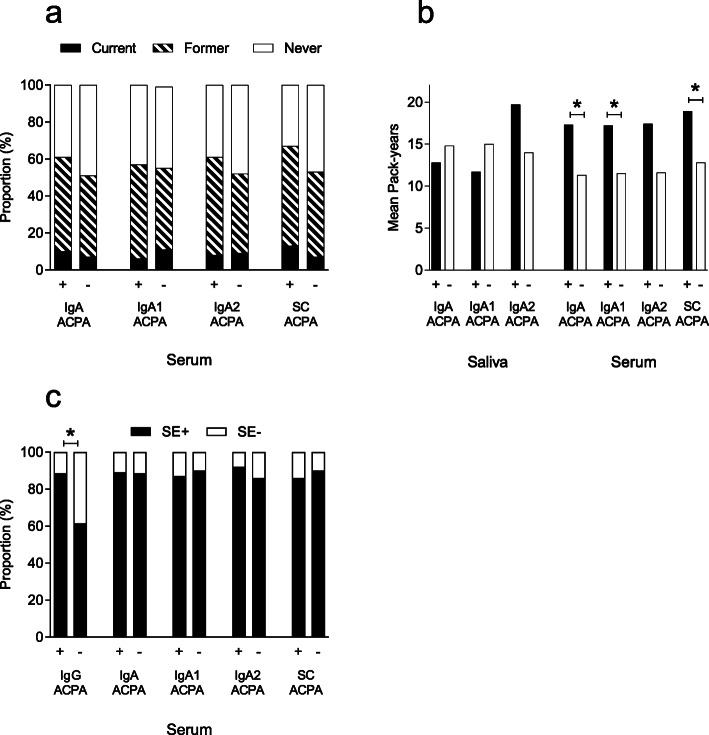
Different ACPAs in relation to **a** smoking status and serum antibodies, **b** cigarette consumption presented as pack-years, and **c** carriage of shared epitope (SE) in different serum ACPAs. *p < 0.05

None of the salivary ACPAs was associated with smoking status (data not shown) or pack-years (Fig. 4b).

#### Serum

Smoking status did not differ significantly according to the status of serum IgA ACPA (including subclasses) or SC ACPA (Fig. 4a). However, the number of pack-years among ever smokers were higher regarding serum IgA, IgA1, and SC ACPA, but not for IgA2 ACPA (Fig. 4b). As expected, serum IgG ACPA-positive patients had higher proportions of SE-positivity compared to IgG-negative patients (89% vs. 62%, *p* < 0.001). When only IgG ACPA-positive patients were included in the analyses, no difference in SE carriage was detected according to serum status of IgA, IgA1, IgA2, or SC ACPA (Fig. 4c).

## Discussion

To our knowledge, this is the largest study to date addressing salivary ACPAs in RA. We characterized the IgA ACPA and IgA ACPA subclass response in paired saliva and serum samples and investigated possible clinical implications. Our main findings are that IgA ACPA responses in the oral cavity as well as in the circulation associate with a more clinically active disease.

In our previous pilot study, IgA ACPA in the saliva occurred in 22% of RA patients and was associated with signs of a milder disease [6]. The present study is the first to confirm the occurrence of salivary ACPA in a subgroup of RA patients, yet the occurrence of IgA ACPA was now lower (12%) in the current, much larger, study population. We could not corroborate the previously suggested negative association between radiographic joint erosions and IgA ACPA in the saliva [6]. Instead, this larger study shows that patients positive for salivary IgA ACPA have higher disease activity at the time of sampling also when considering disease duration and treatment. Furthermore, IgA ACPA levels in the saliva appeared more robustly associated with disease activity than serum levels. Taken together, this suggests that oral mucosal immune responses to citrullinated proteins may trigger effector mechanisms in the RA pathogenesis. In the saliva, ACPA of IgA1 and IgA2 subclasses were uncommon, which hampered further analyses concerning clinical characteristics and RA risk factors.

In serum, IgA1 ACPA was detected in 44% and IgA2 ACPA in 39% of the patients. A previous study on circulating RF IgA subclasses by Otten et al. found circulating IgA1 RF (73%) to be twice as common as IgA2 RF (36%) in RA [23]. Speculatively, our results may suggest that mucosal immunization to citrullinated proteins are particularly relevant in compartments with pronounced IgA2 dominance, such as the lower gastrointestinal tract [12, 28]. Although total IgA2 in serum has recently been found to be more pro-inflammatory than IgA1 in vitro [29], we could not see any difference between serum IgA1 ACPA and IgA2 ACPA regarding their association to disease activity. In the saliva, the reverse was noticed with higher disease activity among IgA1 ACPA-positive patients.

Circulating SC ACPA has in previous studies been found to associate with increased ESR and CRP levels in early RA [17, 18]. Also, in this population of established RA patients, serum SC ACPA is associated with increased disease activity as well as functional disability. However, the correlations between SC ACPA in the serum and IgA ACPA in the saliva were weak and lower than previously reported concerning the lungs and serum SC ACPA [17]. We expected a stronger correlation between circulating SC ACPA and salivary IgA ACPA, as SC ACPA in the circulation supposedly reflects the occurrence of ACPA at mucosal linings. Circulating antibodies containing the SC can be of IgA or IgM isotype, and regarding SC ACPA, IgM has been shown to be the most abundant isotype [30]. Also, free SC correlates more to IgM than does IgA ACPA [31], which could also be an explanation for the weak correlation seen in this study.

Cigarette smoking, causing local irritation of respiratory mucosa, is a possible inducer of ACPA production in RA [24] and associates primarily with mucosa-associated ACPAs [18, 20]. Citrullinated proteins are present in the oral cavity, as shown by immunohistochemical studies on gingival tissue biopsies. The expression of citrullinated proteins appears increased in inflamed tissue [32–34], but the association with smoking was either absent [34] or not investigated [32, 33]. In the present study, there was no association between smoking and IgA ACPA in the saliva, and we could not confirm our hypothesis that IgA2 ACPA would have a stronger association than IgA1 ACPA to smoking. Concerning serum antibodies, there were associations with pack-years, but not with smoking status. The low proportion of current smokers may have contributed to this.

The strengths of this study are the large number of included patients and the paired saliva and serum samples. A limitation is that the radiographic outcome was based on written reports from radiologists, but not subjected to formal scoring. This approach obviously reduces the level of detail, but we still regard that clinically relevant erosive disease could be identified in this way. Also, the cross-sectional design introduces the risk that treatment attenuated possible clinical differences and possibly influenced antibody levels. Finally, the lower mean age among controls may have influenced IgA cutoff levels, as it may associate with age in non-RA patients [35].

For future directions, longitudinal studies are preferred to investigate the possible predictive value of IgA ACPAs in the saliva and serum.

## Conclusion

This large cross-sectional study confirms the presence of salivary IgA ACPA in a small subset of RA patients, in association with a higher disease activity and functional impairment. This suggests that mucosal ACPA responses in the oral cavity may contribute to disease-promoting processes in RA.

## Supplementary Information

**Additional file 1 : Supplementary Table 1.** Radiographic joint erosions versus ACPA isotypes. Abbreviations: *ACPA* anti-cyclic citrullinated peptides, SC secretory component containing.

## Data Availability

The datasets used and/or analyzed during the current study are available from the corresponding author on reasonable request.

## Abbreviations

ACPA: Anti-citrullinated protein antibodies
CAP: Citrullinated arginine peptide
CCP: Cyclic citrullinated peptide
CRP: C-reactive protein
DAS28: Disease activity score 28
ELISA: Enzyme-linked immunosorbent assay
ESR: Erythrocyte sedimentation rate
HAQ: Health Assessment Questionnaire
OD: Optical density
PGA: Patient global assessment
RA: Rheumatoid arthritis
RF: Rheumatoid factor
SC: Secretory component
SE: Shared epitope (HLA-DRB1)
SIgA: Secretory IgA
SJC: Swollen joint count
TJC: Tender joint count
