# Supplementary material for: Presence of salivary IgA anti-citrullinated protein antibodies associate with higher disease activity in patients with rheumatoid arthritis
Source: Arthritis Res Ther. 2020 Nov 23;22:274. doi: 10.1186/s13075-020-02363-0 (PMC7681967; doi:10.1186/s13075-020-02363-0)
Supplement: Supplementary file 1 — Additional file 1 : Supplementary Table 1. Radiographic joint erosions versus ACPA isotypes. Abbreviations: ACPA anti-cyclic citrullinated peptides, SC secretory component containing. [file 13075_2020_2363_MOESM1_ESM.docx]

**Supplementary table 1. Radiographic joint erosions versus ACPA isotypes.**

|  | **Erosions present** | **No erosions present** | **p-value** |
| --- | --- | --- | --- |
| **Saliva:** |  |  |  |
| IgA ACPA, positive/total (%) | 12/89 (13) | 10/66 (15) | 0.818 |
| IgA1 ACPA, positive/total (%) | 11/88 (13) | 8/66 (12) | 1 |
| IgA2 ACPA, positive/total (%) | 6/88 (7) | 8/66 (12) | 0. 273 |
|  |  |  |  |
| **Serum:** |  |  |  |
| IgA ACPA, positive/total (%) | 50/89 (56) | 36/66 (55) | 0.871 |
| IgA1 ACPA, positive/total (%) | 49/89 (55) | 35/66 (53) | 0.871 |
| IgA2 ACPA, positive/total (%) | 38/89 (43) | 34/66 (52) | 0.329 |
| SC ACPA, positive/total (%) | 22/89 (25) | 19/66 (29) | 0.586 |

Abbreviations: *ACPA* anti-cyclic citrullinated peptides, SC secretory component containing.
